# Dental Society of the State of New York

**Published:** 1885-08

**Authors:** 


### DENTAL SOCIETY OF THE STATE OF NEW YORK.

### SEVENTEENTH ANNUAL MEETING HELD AT ALBANY, MAY, 1885.

(Continued from page 323.)

#### WEDNESDAY EVENING SESSION.

After the reading of the minutes of the previous session, Dr. Chas. F. Ives read a paper upon the subject of "Pulpless Teeth," of which the following is a condensed summary :

I have no intention of going over any portion of the ground so repeatedly and thoroughly trodden by others on this subject, but rather to offer a few thoughts suggested to my mind after reading the articles in the *Medical Record*, and listening to the remarks of different members of the dental profession in reply.

Entirely agreeing with the verdict that physicians generally are far from being well posted in diseases of the teeth, I could not help wondering why these articles were written. It surely was not from any desire to snub us as a profession, nor to gratify any personal antipathy, and they were not altogether the result of clinical observation.

Back of all this there must have been some honest conviction which led to the attack; perhaps a succession of unpleasant experiences, the results of slow and wearisome endeavors to find a cause for certain pathological conditions, the tracing of which through various avenues leading to one common centre, resulted in certain convictions so strong as to cause the author to overlook the fact that, under other conditions than those existing, the results would perhaps have been different. For instance, if in their practice they meet with cases that baffle them in diagnosis and treatment, and after having almost exhausted their skill in a vain attempt to get at the cause they reach the fact that there is at times an occasional approach to pain sufficient to be acknowledged, and if this general uneasiness is at last traced to a pulpless tooth which has been treated and filled, or to one in which an attempt has been made to cap an exposed pulp, or in which a metal filling has been placed too close to it, and if extraction at once relieves all the trouble for the alleviation of which the physician was called, the experience will naturally lead the physician to the conclusion that pulpless teeth are foreign bodies, and that the sooner they are removed the better. I have heard more than one assert experiences like this. Can we blame them? We know that if pulpless teeth are brought to a healthy condition and their roots filled as well as possible, the annoying cases are reduced to a minimum and there is no necessity for consulting a physician regarding any trouble from them.

What proportion of pulpless teeth can be honestly and intelligently treated and filled by the dentist? Of how many of the teeth coming to him can he say that he has done his best, regardless of time or remuneration? This is the serious side. To illustrate:—A gentleman on his way to have the first superior molar extracted, thought better of the idea, and came to me for relief. Having received it, he volunteered the following statement: Some four years before he had put himself in the hands of a New York dentist, who found an exposed pulp in this tooth. It had a large gold filling, but the applications for destruction and treatment were made through a cavity in the posterior approximal surface. In due time the dead pulp was removed, and an attempt made to enter the root canal. The palatal root offered but little difficulty, but the buccal was more of a task. After many trials the dentist told him they were entirely closed with secondary dentine. The palatal root was dressed, and a temporary stopping inserted. From that time on the tooth was a constant source of uneasiness. Some time after, the patient went to another dentist, who drilled through the crown filling, obtaining direct access to the root. Treatment followed with but little relief, and it was finally filled with gutta-percha. This was the history when he came to me. I removed the entire filling, and enlarged the opening until I had free access to all the roots. I found the entrance to both buccal roots, and followed them until the freedom from odor convinced me there would be no more trouble. The palatal root was the seat of the whole di-tress. It was stubborn under treatment, and Carbolic acid or Iodoform left in it for a few days would lose identity. I went through the foramen with a drill made from a Donaldson bristle. A discharge of pus followed, and from that time it has continued to improve, and I hope will be soon in a condition to fill permanently. Well, what of it? Simply this. Do you honestly believe that if the dentist who destroyed the pulp had removed the crown filling and given the roots proper treatment and filled them, it would have given trouble? Could we look for success from treatment in any other way? It is such imperfect operations that send not a few to the physician with suspected neuralgia and other nervous diseases. They naturally result in the reporting of such operations in medical journals, with an editorial at the end, to the effect that "dentists should remember that the treatment of diseased tissues requires medical education."

It needs a great deal of patience and good temper to treat a refractory pulpless tooth. No operation so poorly pays. No operation draws more heavily on the nervous system, for it discourages as well as wearies, and it is at such times that the tempter draws near, and one needs all his moral strength to say, "Get thee behind me." If we are unwilling to give our best efforts, our greatest energy to the work, to devote the time necessary, then let us believe with the physicians that extraction is the best thing, and not pity their ignorance. If, on the other hand, we strive with honesty of purpose to do the best we can, we shall prove in a large degree successful, and show our medical friends that pulpless teeth can be made respectable members of oral society, even though they are slightly crippled.

The essayist then spoke of the different materials used for root fillings, stating that he never yet, in the removal of a filling, found any of gutta-percha, metal of any kind, wood or paste, that did not give forth a vile odor. He claimed that, so far as adaptation is concerned, a larger proportion could be perfectly filled with a solution of gutta-percha and chloroform than with any other material, but that it could not be made impervious to emanations. He had occasion to remove the filling and refill a superior bicuspid, the roots of which had been filled with cotton and creosote thirteen years before. As each portion of the filling was removed it was passed close to the nasal organ, and found redolent of the antiseptic. He, therefore, put it down that cotton and creosote can be made to answer a good purpose. In his hands nothing has been so uniformly successful as a slow setting oxy-chloride of zinc, mixed to a moderate stiffness, carried to position on a fibre or two of cotton.

He has no sympathy with the practice that proceeds upon the principle that all root fillings should be tentative in character, put in place in the expectation of removal if it becomes necessary, or with that of providing a tap or vent hole. He would rather direct his energies to filling them as well as any other cavity.

*Dr. Rhein*—The admirable points in the paper just read have been, no doubt, understood by every one. There is only one thing of which I wish to speak, and that is in regard to what has been said about gutta-percha fillings in the root. I believe a root can be perfectly filled with gutta-percha, and give no trouble and no odor. I have removed gutta-percha fillings and never detected any odor. It depends a great deal upon the manner in which the tooth is filled. It is more satisfactory than anything I have ever used. With gutta-percha I can reach the very apex. After becoming convinced that there is no trace of putrefaction left, I introduce a little alcohol into the cavity. Having my alcohol lamp and hot air syringes handy, I dry the root out very thoroughly, so that it is warm, and then introduce the gutta-percha solution upon a platinum point. I fill up half the root in that way. I then fill the tooth with oxy-phosphate, which I prefer to the oxy-chloride, because it is easier to handle. I do not mean to say that no other filling will perfectly fill a root. I presume there are a hundred ways of filling them successfully, but what I assert is that gutta-percha fillings will preserve roots perfectly without taint.

*Dr. Ives*—I believe I stated that any filling could be used so perfectly that not the slightest odor could be detected at the time, but it will be offensive at the end of the year. You cannot make a gutta-percha filling that will not absorb, and if it does it will have an odor.

*Dr. Rhein*—I would like to inquire where the absorption could come from if you have a hermetically sealed cavity. I do not propose to say that imperfectly filled teeth will not act so, but I am speaking of an ideal operation.

On motion, the subject was passed. The Herbst system of filling teeth was the subject next called, and Dr. Bodecker delivered the opening address. (See page 395).

Dr. Frank French moved that Dr. Herbst be elected an honorary member of the Society.

The motion was carried unanimously.

Dr. Wm. H. Waite, of Liverpool, England, was then introduced to the Society, and read a paper upon events connected with the dental profession in England. (See page 339, July number).

After the reading of his paper, Dr. Waite presented a resolution from the Midland Branch of the British Dental Association, extending their kindly greetings to the Dental Society of the State of New York.

Dr. Hill moved that the Dental Society of the State of New York receive with great pleasure the expression of the fraternal feeling of our English professional friends, and that the Secretary be instructed in return to convey to the Midland Branch of the British Dental Association our cordial salutations, and to express the hope that through the mediumship of their Secretary, who is also an esteemed honorary member of this Society, the personal and professional relationship between the members of the two bodies may become and ever remain a close and intimate one.

Dr. S. B. Palmer, of Syracuse, then spoke upon the subject of the discoloration of gold fillings. He referred to the various articles upon the subject in the journals, and said that it was desirable that the matter be better understood. He related various experiments which he had made, from which he was fully convinced that the most frequent cause of discoloration was iron. It was his custom, he remarked, when removing such fillings, to inquire of the patient the kind of food used, and it was usually found that it had been prepared in iron vessels. Iron is frequently given as a tonic, and inquiry will show that such patients frequently have discolored fillings. His theory was that the iron was deposited by galvanic action, and that the gold was extremly apt to collect it.

Adjourned till 9.15 o'clock.

May 14th—Morning Session.

The meeting was called to order, and the minutes of the last evening's session read and approved.

The committee to draft resolutions upon the death of Dr. J. G. Ambler presented their report.

After the transaction of the usual routine business, the regular order was called, and Dr. Frank Abbott, according to announcement, spoke upon "Dental Therapeutics." He stated that the subject was one so enormously large that it was impossible to give more than a sketch, and he could refer to but a few of the remedies used. After speaking upon the theories concerning the decay of the teeth, the use of antiseptics, and the treatment of diseases of the mouth, he referred to the different remedies used and the effects desired to be produced. Muscular rheumatism was almost entirely removed by the use of antacids. Bicarbonate of potash, given in from ten to twenty grain doses two or three times a day, would relieve it. Salicylate of soda would do the same. When taken, it seems to have a peculiar effect upon the teeth, relieving them of sensitiveness. Carbonate and muriate of magnesia are also valuable antacid remedies. The latter is very harmless, and if taken in tablespoon doses is a laxative. Rochelle salts is an excellent remedy, and sometimes has a wonderful effect within twenty-four hours. Lime water is another very pleasant remedy. Where teeth are so sensitive that they cannot be touched, if this be given, it is not improbable that the next day you can excavate without fear of pain. I might instance also bicarbonate of soda. I have never used a remedy that gave so much comfort to my patients as this, not even cocaine. Perhaps the best way to administer it is in compressed pellets.

Bichloride of mercury is the simplest antiseptic we have. In from 1 to 20,000 parts of water it will destroy septic-organisms. Use half a grain to twenty-one ounces of water. Iodoform and carbolic acid are very useful remedies, especially the latter.

Boracic acid, carbonate of soda, permanganate of potash, listerine, and other remedies were recommended. He frequently uses them in the form of a spray, thrown into the mouth from an atomizer.

To show the comparative antiseptic properties of the different remedies, he read from a list found in the book known as "Treatment of Wounds," by L. S. Pilcher, M. D., giving the advisable strength of the solution of the different antiseptics.

"Elements of Decay" was the next subject, and Dr. Barrett was called upon to open the discussion. This, he said, was such an extensive subject that he would only attempt to allude to fermentation and putrefaction. Decay was co-extensive with creation. In the language of Dr. Atkinson, you cannot conceive of the "togetherness" of atoms without their "apartness." If brought together, it is because they were separated. If divided, it is because they were once together.

During the past few years the subject of micro-organisms has engaged the attention of some of the best minds of the human race. The infinitely small transcends the infinitely great. The micro-cosmos is infinitely greater than the macro-cosmos. We cannot comprehend it.

I draw a clear line of demarkation between fermentation and putrefaction. They are separate processes, though tending to the same end; they are produced by different means, and their action is essentially different. So far as I know, putrefaction is always brought about by micro-organisms, while fermentation sometimes is not. Fermentation is a disruption of cohesion. How this occurs we know not. Putrefaction is a decay of the atoms themselves. The two processes cannot exist together. Putrefactive organisms work upon nitrogenous matter, while the fermentative organisms do not.

The successful introduction of putrefactive germs in a fermentation or fermentative compound arrests that process. Let me instance the making of bread, or beer. Putrefaction means the entire spoiling of the fermentative production. Fermentation is largely the process of converting starch into sugar, and albumen into peptone.

As I understand putrefaction, it is the rapid and constant disruption of an organic substance, through the agency of fungi. Fermentation results in the production of a new substance, not necessarily through the action of fungi. One is destructive, and the other, although destructive, is at the same time constructive.

Dr. Bogue spoke upon the subject of Amalgam Fillings, referring to the various papers that have been published since 1882, especially mentioning those of Drs. Hitchcock, Talbot and Collier.

Amalgam fillings are found in the mouth uncorroded by iron or acid drinks. Sulphur collects upon amalgams, and blackens them, but it is from the effect of the acid. If amalgams are inserted with a good deal of mercury and a powerful pressure, it brings the mercury to the surface, and thence it may be carried to the stomach. I have read, within the past two months, of something like two pounds of mercury being put into the stomach by a tube, and in a few days being discharged, and the patient recovered. I think we may safely assert that metallic mercury is harmless, unless it is converted into an oxide or chloride.

Dr. W. H. Waite exhibited to the Society specimens of false teeth connected by bridge work, found in an Etruscan grave, and which were pre-historic. He obtained them from the museum at Liverpool, after much trouble and correspondence.

The specimens were very interesting, and formed the subject of considerable discussion among the members present. It was voted that the thanks of the Society be extended to Dr. Waite for his trouble and kindness in bringing these curious specimens, and also to the proprietor of the museum for his courtesy in allowing him to do so.*

Dr. Atkinson then read a paper upon the subject of "Sponge Grafting." (An abstract of this paper will appear hereafter.—Ed.)

Permission was given the publishers of the Independent Practitioner to make abstracts from the reports and papers of the Society for publication.

It was suggested by Dr. Baxter that steps be taken to obtain rooms in the new Capitol, in which to hold the annual sessions of the Society, and a committee was appointed to look the matter up.

Routine business followed, after which the following officers were elected for the ensuing year:

President, Dr. F. B. Darby, of Elmira.

Vice-President, Dr. G. C. Daboll, of Buffalo.

Secretary, Dr. J. Edward Line, of Rochester.

Treasurer, Dr. H. G. Mirick, of Brooklyn.

Corresponding Secretary, Dr. W. H. Atkinson, of New York.

First District Censor, Dr. Wm. Carr, of New York.

Third District Censor, Dr. S. D. French, of Troy.

The newly-elected President was conducted to the chair by Drs. Hill and F. French, and introduced to the Society by the retiring President, Dr. Straw. He thanked the Society for the honor conferred upon him, and asked the pleasure of the meeting.

Upon motion of Dr. Atkinson, Dr. W. B. Hailes, Jr., was elected a corresponding member. Upon motion of Dr. Line, Dr. Alex.

---

* The specimens exhibited by Dr. Waite were the ones which formed the subject of his article in the number of this Journal for April, 1885, and were similar to the ones forming the subject of the illustrations to Dr. Van Marter's paper in the number for January.—Editor.

Campbell, of British Columbia, was also made a corresponding member.

After the appointment of committees on Arrangements, Publication, and Dental Law, the Society adjourned.

